# High miR203a-3p and miR-375 expression in the airways of smokers with and without COPD

**DOI:** 10.1038/s41598-022-09093-0

**Published:** 2022-04-04

**Authors:** Jos van Nijnatten, Corry-Anke Brandsma, Katrina Steiling, Pieter S. Hiemstra, Wim Timens, Maarten van den Berge, Alen Faiz

**Affiliations:** 1 Department of Pathology and Medical Biology, University Medical Center Groningen, The University of Groningen, Groningen, The Netherlands; 2 Department of Pulmonary Diseases, University Medical Center Groningen, The University of Groningen, Groningen, The Netherlands; 3 Groningen Research Institute for Asthma and COPD, University Medical Center Groningen, The University of Groningen, Groningen, The Netherlands; 4 Respiratory Bioinformatics and Molecular Biology, The University of Technology Sydney, Sydney, NSW Australia; 5 Bioinformatics Program, Boston University, Boston, MA USA; 6 Section of Pulmlonary and Critical Care Medicine, Boston University School of Medicine, Boston, MA USA; 7 Department of Pulmonology, Leiden University Medical Center, Leiden, The Netherlands

**Keywords:** Epigenetics, Risk factors

## Abstract

Smoking is a leading cause of chronic obstructive pulmonary disease (COPD). It is known to have a significant impact on gene expression and (inflammatory) cell populations in the airways involved in COPD pathogenesis. In this study, we investigated the impact of smoking on the expression of miRNAs in healthy and COPD individuals. We aimed to elucidate the overall smoking-induced miRNA changes and those specific to COPD. In addition, we investigated the downstream effects on regulatory gene expression and the correlation to cellular composition. We performed a genome-wide miRNA expression analysis on a dataset of 40 current- and 22 ex-smoking COPD patients and a dataset of 35 current- and 38 non-smoking respiratory healthy controls and validated the results in an independent dataset. miRNA expression was then correlated with mRNA expression in the same patients to assess potential regulatory effects of the miRNAs. Finally, cellular deconvolution analysis was used to relate miRNAs changes to specific cell populations. Current smoking was associated with increased expression of three miRNAs in the COPD patients and 18 miRNAs in the asymptomatic smokers compared to respiratory healthy controls. In comparison, four miRNAs were lower expressed with current smoking in asymptomatic controls. Two of the three smoking-related miRNAs in COPD, miR-203a-3p and miR-375, were also higher expressed with current smoking in COPD patients and the asymptomatic controls. The other smoking-related miRNA in COPD patients, i.e. miR-31-3p, was not present in the respiratory healthy control dataset. miRNA-mRNA correlations demonstrated that miR-203a-3p, miR-375 and also miR-31-3p expression were negatively associated with genes involved in pro-inflammatory pathways and positively associated with genes involved in the xenobiotic pathway. Cellular deconvolution showed that higher levels of miR-203a-3p were associated with higher proportions of proliferating-basal cells and secretory (club and goblet) cells and lower levels of fibroblasts, luminal macrophages, endothelial cells, B-cells, amongst other cell types. MiR-375 expression was associated with lower levels of secretory cells, ionocytes and submucosal cells, but higher levels of endothelial cells, smooth muscle cells, and mast cells, amongst other cell types. In conclusion, we identified two smoking-induced miRNAs (miR-375 and miR-203a-3p) that play a role in regulating inflammation and detoxification pathways, regardless of the presence or absence of COPD. Additionally, in patients with COPD, we identified miR-31-3p as a miRNA induced by smoking. Our identified miRNAs should be studied further to unravel which smoking-induced inflammatory mechanisms are reactive and which are involved in COPD pathogenesis.

## Introduction

Smoking is one of the leading causes of preventable death worldwide, and despite the well-known health effects, over 15% of the world’s population still smoked in 2019, according to the WHO. The smoking of cigarettes exposes the lung to more than 4000 components^1^. As a result, it is strongly associated with the development of lung cancer^2,3^ and other respiratory diseases^4,5^, including chronic obstructive pulmonary disease (COPD). Characteristics of COPD are an inflammatory response in the airways and lung parenchyma, associated with progressive, irreversible airflow limitation, dyspnea, hypersecretion of mucus, and alveolar destruction, i.e., emphysema.

We have previously shown that current smoking affects levels of gene expression and DNA methylation in bronchial biopsies^6^. However, current smoking on microRNA expression in the human airway wall is not well studied so far^7,8^. MicroRNAs (miRNA) are small non-coding RNA transcripts (approximately 17–27 nucleotides long), which interact with the Argonaute protein (AGO protein) in the RNA‐induced silencing complex (RISC), leading to cleavage or silencing of a target mRNA^9^. Most human protein-coding genes contain miRNA target sites^10^. A single miRNA can influence the expression of hundreds of genes^11^ and regulate a broad range of biological processes, such as cell proliferation, apoptosis, and the immune system^12,13^. Because of this, deregulation of miRNA function is associated with numerous diseases, including COPD^14–16^.

We also have previously shown that miRNAs play an essential role in mediating inflammatory responses and corticoid effects in asthma and COPD^17–20^. However, although several studies have assessed the impact of smoking on miRNA expression, very few have focused on the airway wall in an unbiased method^21^. Here, we investigated the influence of current smoking on miRNA expression in bronchial biopsies in two independent datasets consisting of current and ex-smoking COPD patients and current and non-smoking respiratory healthy controls to elucidate the overall smoking-induced miRNA changes and those specific to COPD. In addition, we correlated miRNA to matched mRNA expression to identify genes and pathways influenced by the miRNAs affected by smoking to investigate the downstream effects on gene expression. Finally, to investigate the relation with cellular composition, differential miRNAs expression was associated with gene signatures of specific cell populations and immunohistological staining of specific cell types in matched biopsies.

## Methods

### Subjects

Affymetrix GeneChip miRNA 1.0 Array and RNA-Seq data from baseline bronchial biopsies from COPD patients and respiratory healthy controls were used for this study as described previously^22,23^.

COPD patients participated in the Groningen Leiden Universities and Corticosteroids in Obstructive Lung Disease study (GLUCOLD, ClinicalTrails.gov NCT00158847) (n = 62). Respiratory healthy controls participated in the Study to Obtain Normal Values of Inflammatory Variables from Healthy Subjects (NORM study, ClinicalTrails.gov NCT00848406, n = 73). In- and exclusion criteria and more extensive characteristics of subjects participating in the GLUCOLD and NORM studies were previously described^24,25^.

Briefly, for the GLUCOLD study, all patients were current or ex-smokers with > 10 pack years. Participants had irreversible airflow with postbronchodilator forced expiration volume in one second (FEV_1_) < 80% predicted and postbronchodilator FEV_1_/forced vital capacity (FVC) below 70%. Patients took no oral corticosteroids for at least three months before starting the study and did not use inhaled corticosteroids for at least six months prior to the study. Patients classified as ex-smokers did not smoke at least one year prior to the study.

Participants of the NORM study had normal lung function (FEV_1_ > 80% and FEV_1_/FVC > 70%) and no bronchial hyperresponsiveness to methacholine (PC_20_ > 16 mg/ml). Participants defined as non-smokers did not smoke during the year leading up to the study and did not smoke more than one year in total, with a total of < 0.5 packyears.

The local medical ethics committees approved all studies, and all subjects gave their written informed consent (the NORM study was approved by the ethics committee of the UMCG, and the GLUCOLD study was approved by the same committee and the ethics committee of the LUMC)^24–26^. All methods were performed in accordance with the relevant guidelines and regulations.

### Statistics

To identify differentially expressed miRNAs in bronchial biopsies of current and ex-smoking COPD patients and current and non-smoking healthy participants, we used a linear model using Limma (R-package version 3.40.6, R statistical software version 3.6). We used current smoking status as a categorical variable, adjusting for age and sex. A Benjamini–Hochberg corrected p-value (False Discovery Rate (FDR)) of < 0.05 was considered statistically significant.

We performed the identification of potential targets of differentially expressed miRNAs by directly correlating miRNA expression to normalized^27^ gene expression data available from the same bronchial biopsies. We correlated significant differentially expressed miRNAs to all expressed mRNAs using the Pearson Correlation Coefficient. Significant correlations between miRNA expression levels and gene expression levels were determined using Psych (R-package version 1.8.12), and we performed a meta-analysis on the results of both studies. Significant correlations with an absolute R-value above 0.25 were considered biologically relevant.

We verified the smoking-related expression changes of the identified miRNAs using an independent miRNA dataset (Illumina HiSeq) obtained from bronchial brushings from 30 COPD patients and 30 non-COPD controls^28^. COPD was defined in this dataset by FEV_1_/FVC < 70% and FEV_1_ < 80% predicted and non-COPD controls were defined as FEV_1_/FVC >  = 70 or FEV_1_/FVC < 70% and FEV_1_% predicted > 80%. We used a linear model and smoking status as a categorical variable while correcting for age, sex and pack years. We analyzed the COPD and non-COPD participants separately.

### miRNA predicted target approach

We calculated the most likely target for each miRNA using miRNAtap (R-package version 1.18.0). The default settings were used to calculate the geometric mean of ranks from the databases/algorithms PicTar, Diana, TargetScan, miRanda, and miRDB. The minimum number of sources required for a potential target was 2.

### Pathway analysis

To predict which pathways are influenced by smoking-induced miRNAs, we performed Gene Set Enrichment Analysis (GSEA version 4.0.3) on the mRNAs positively and negatively correlated with our differentially expressed smoking-associated miRNAs. A separate analysis was done for the negatively correlated predicted target genes only. We compared the ranked lists to BioCarta and KEGG gene set databases. We then performed a meta-analysis on the pathways found in both studies and adjusted the p-value using Benjamini-Hochberg's method.

### Association of miRNAs with composite scores of cell-type-specific genes based on GSVA

We used cell-type-specific gene signatures based on single-cell data for 15 cell types (activated endothelium, B-cells, basal cells, proliferating basal cells, ciliated cells, club cells and goblet cells, fibroblasts, inflammatory dendric cells, ionocytes, luminal macrophages, mast cells, neutrophils, smooth muscle cells, and submucosal cells)^29^. We then performed Gene Set Variation Analysis (GSVA^30^, R-package version 1.32.0) to calculate the composite scores for each cell type. We then correlated each composite score to relevant miRNAs using the Pearson correlation coefficient.

### Evaluation of miRNAs with the presence of cell populations in matched bronchial biopsies

For the differentially expressed miRNAs, we assessed the correlation between miRNA expression and numbers of eosinophils, neutrophils, macrophages, mast cells, CD3 or CD4 or CD8 positive T-cells, and percentage of Periodic acid–Schiff stain (PAS)-positive goblet cells in paraffin-embedded bronchial biopsies from the same patients taken at the same location in the lung. These data were obtained from previously published data sets for the GLUCOLD^31^ and NORM^26^ studies.

**Figure 1:**
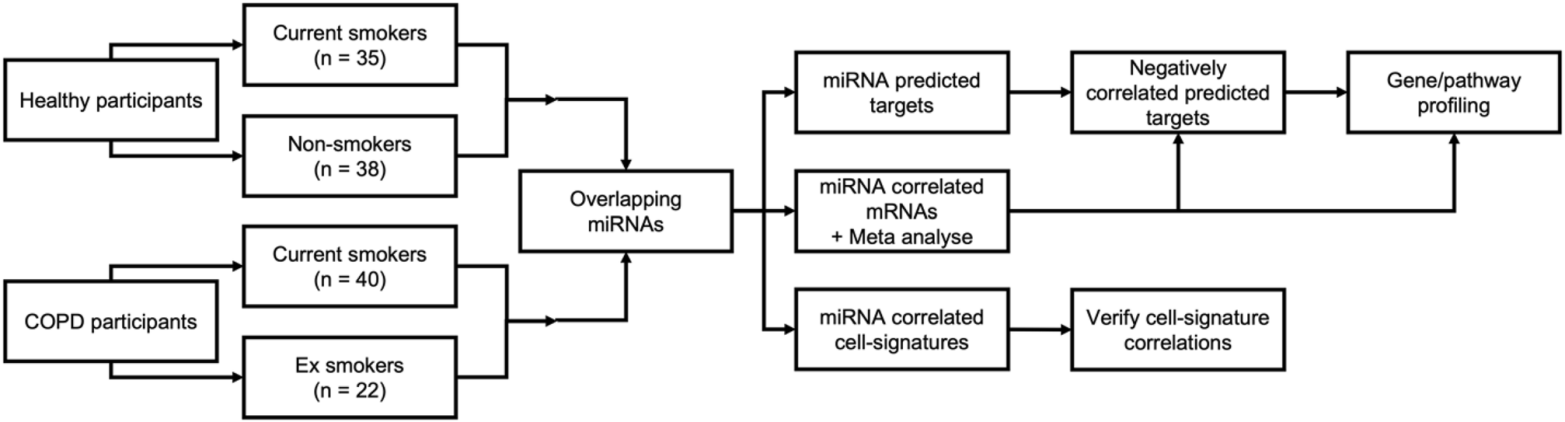
Flow diagram of the study approach. Two studies were used: the GLUCOLD study with current or ex-smoker COPD patients and the NORM study with current or non-smoking respiratory healthy controls. A linear regression model was used to identify smoking-associated miRNAs. The overlapping two miRNAs were used for further analysis of COPD patients. A meta-analysis of Pearson correlations was performed to identify miRNA associated mRNA. The predicted targets were obtained, and a list of negatively correlated predicted targets was compiled. Pathway analysis was performed using this list as well as on all the negative and positively correlated mRNAs. Negatively correlated predicted targets were repeated in the NORM. Additionally, miRNA expression was correlated to cell type signatures and cell populations.

A flow diagram showing the analysis approach is provided in Fig. 1.

## Results

In the current study, we investigated smoking-related miRNA changes in 62 COPD patients (ex-smokers, n = 22, and current smokers, n = 40) and 73 respiratory healthy individuals (non-smokers, n = 38 and current smokers, n = 35). The subject characteristics of the two studies are presented in Table 1. There was a significant difference in sex between the COPD patients and respiratory healthy individuals (Chi-Squared p-value = 3E−4), as well as a significant difference in age (Mann–Whitney U p-value = 2E−11).

**Table 1:** Patient demographics in relation to smoking status.

	COPD patients; GLUCOLD		Respiratory Healthy controls; NORM	
	Ex-smokers	Current smokers	Non-smokers	Current smokers
N	22	40	38	35
Age mean ± SD	63 ± 8	59 ± 7	38 ± 19	41 ± 15
Sex male n (%)	20 (90.9)	33 (82.5)	20 (52.6)	20 (57.1)
FEV_1_% predicted	55.9 ± 10.7	54.7 ± 9.5	101.4 ± 12.2	99.1 ± 9.4
FEV_1_/FVC %	50.1 ± 9.3	46.8 ± 8.5	80.5 ± 6.6	78.0 ± 6.2
Age quit smoking mean ± SD	56 ± 10			
Cigarettes per day mean ± SD	0	18.5 ± 10.2	0	15.0 ± 7

*FEV*_*1*_ forced expiratory volume in 1 s, *FVC* forced vital capacity.

### Changes in miRNA expression associated with current smoking

**Figure 2:**
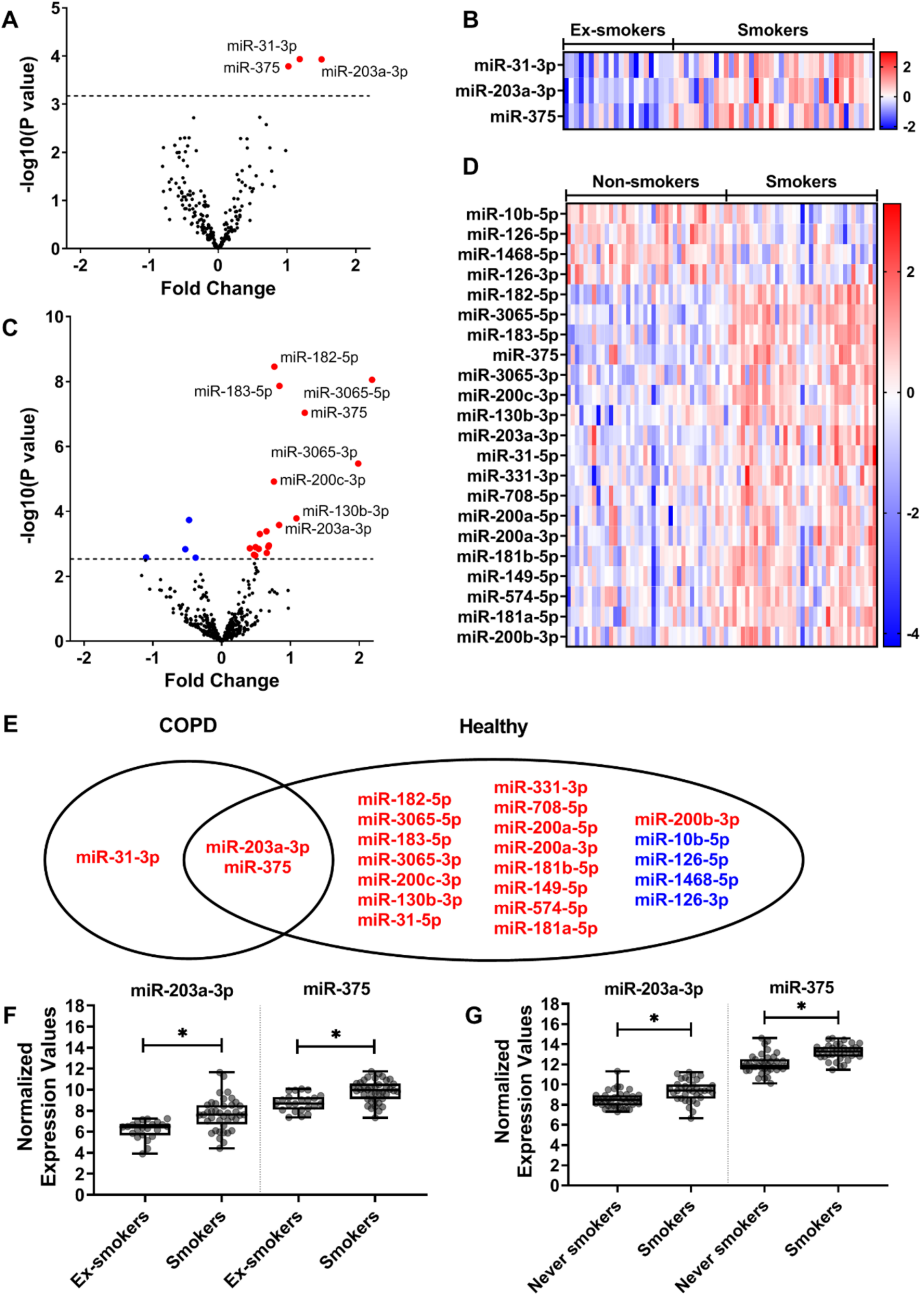
Differentially expressed microRNAs (miRNAs) when comparing smoking status in patients with COPD and respiratory healthy controls. Volcano plot (**A**) shows changes in expression levels of 220 miRNAs in patients with COPD (n = 62). Heat map (**B**) shows expression changes of the three significantly differentially expressed miRNAs in COPD patients. Volcano plot (**C**) shows changes in the expression of 395 miRNAs in respiratory healthy controls (n = 73). Heat map (**D**) shows expression changes of the 22 significantly differentially expressed miRNAs in respiratory healthy controls. Venn diagram (**E**) shows the overlap between significantly differentially expressed miRNAs in COPD patients compared to respiratory healthy controls. Boxplot (**F**) shows the normalized expression values of the miRNAs miR-203a-3p and miR-375 per smoking status group in the COPD patients, and boxplot (**G**) shows the normalized expression values for the same miRNAs in the respiratory healthy controls. Red indicates an increase in expression, whereas blue indicates a decrease in expression. A false discovery rate (FDR) cut-off of 0.05 was used.

We identified three miRNAs (miR-31-3p, miR-203a-3p, and miR-375) differentially expressed between current- and ex-smoking COPD patients (FDR < 0.05). All three miRNAs showed a higher level of expression in current- compared to ex-smokers (Table 2). A volcano plot and heatmap are depicted in Fig. 2A,B.

**Table 2:** Differential expression of miRNAs for respiratory healthy controls and subjects with COPD when comparing smoking status.

miRNA id	COPD			Asymptomatic		
	Log_2_(FC)	P-value	FDR	Log_2_(FC)	P-value	FDR
hsa-miR-31-3p	1.18	1.16E−04	1.21E−02	NA	NA	NA
hsa-miR-203a-3p	1.50	1.17E−04	1.21E−02	0.83	2.63E−04	1.15E−02
hsa-miR-375	1.02	1.64E−04	1.21E−02	1.21	9.22E−08	9.10E−06
hsa-miR-200b-3p	0.70	2.65E−03	9.80E−02	0.49	2.28E−03	4.50E−02
hsa-miR-183-5p	0.75	8.10E−03	1.07E−01	0.84	1.37E−08	1.80E−06
hsa-miR-31-5p	0.46	1.96E−02	1.68E−01	0.65	4.10E−04	1.62E−02
hsa-miR-200c-3p	0.35	2.40E−02	1.96E−01	0.76	1.21E−05	7.94E−04
hsa-miR-200a-5p	0.55	3.06E−02	2.27E−01	0.68	1.22E−03	3.38E−02
hsa-miR-182-5p	0.35	4.16E−02	2.58E−01	0.76	3.50E−09	1.38E−06
hsa-miR-200a-3p	0.32	6.45E−02	2.87E−01	0.49	1.26E−03	3.38E−02
hsa-miR-708-5p	0.49	6.51E−02	2.87E−01	0.69	1.12E−03	3.38E−02
hsa-miR-149-5p	− 0.35	1.79E−01	4.32E−01	0.54	1.43E−03	3.38E−02
hsa-miR-181b-5p	0.15	3.65E−01	6.31E−01	0.41	1.37E−03	3.38E−02
hsa-miR-574-5p	0.24	4.21E−01	6.54E−01	0.65	1.91E−03	4.18E−02
hsa-miR-331-3p	0.14	4.79E−01	6.94E−01	0.55	4.96E−04	1.78E−02
hsa-miR-130b-3p	0.11	5.04E−01	7.22E−01	1.09	1.64E−04	9.06E−03
hsa-miR-181a-5p	0.05	7.70E−01	8.81E−01	0.47	2.17E−03	4.50E−02
hsa-miR-126-3p	− 0.03	8.99E−01	9.45E−01	− 0.38	2.65E−03	4.76E−02
hsa-miR-10b-5p	− 0.01	9.81E−01	9.91E−01	− 0.47	1.83E−04	9.06E−03
hsa-miR-3065-5p	NA	NA	NA	2.18	8.79E−09	1.74E−06
hsa-miR-3065-3p	NA	NA	NA	1.98	3.36E−06	2.66E−04
hsa-miR-126-5p	NA	NA	NA	− 0.53	1.45E−03	3.38E−02
hsa-miR-1468-5p	NA	NA	NA	− 1.10	2.62E−03	4.76E−02

*FC* fold change, *FDR* false discovery rate (Benjamini Hochberg corrected p-value).

Differentially expressed miRNAs with an FDR < 0.05 are shown. The table is sorted by the P-value of the COPD participants. NA's are given for probes not available in the dataset.

We next repeated this analysis in respiratory healthy individuals. A total of 22 miRNAs were differentially expressed in the current- versus non-smokers (FDR < 0.05). Of these, 18 miRNAs showed a higher expression level in current smokers than non-smokers, while four were lower expressed (Table 2). A volcano plot and heatmap are depicted in Fig. 2C,D, respectively.

Two miRNAs, miR-375 and miR-203a-3p, were associated with current smoking in the same direction in both COPD patients and respiratory healthy controls (Fig. 2E–G).

MiR-31-3p, the third smoking-associated miRNA in the COPD patients, was not present in the respiratory healthy small-RNA-seq dataset.

We validated our findings in an independent dataset of epithelial brushes. We confirmed higher miR-375 expression in current- than ex-smokers in COPD and non-COPD, but no effect of smoking status on miR-203a-3p or miR-31-3p. (Supplementary Table S4).

### Associations between changes in miRNA expression and mRNA expression

To identify potential genes and pathways that are regulated by the three smoking-associated miRNAs, we used two different methods: (1) we investigated the global effects of the miRNAs by doing direct correlations with all expressed genes in the same biopsies, and (2) we investigated the direct effects by focusing on the negatively correlated predicted targets for each miRNA.

**Figure 3:**
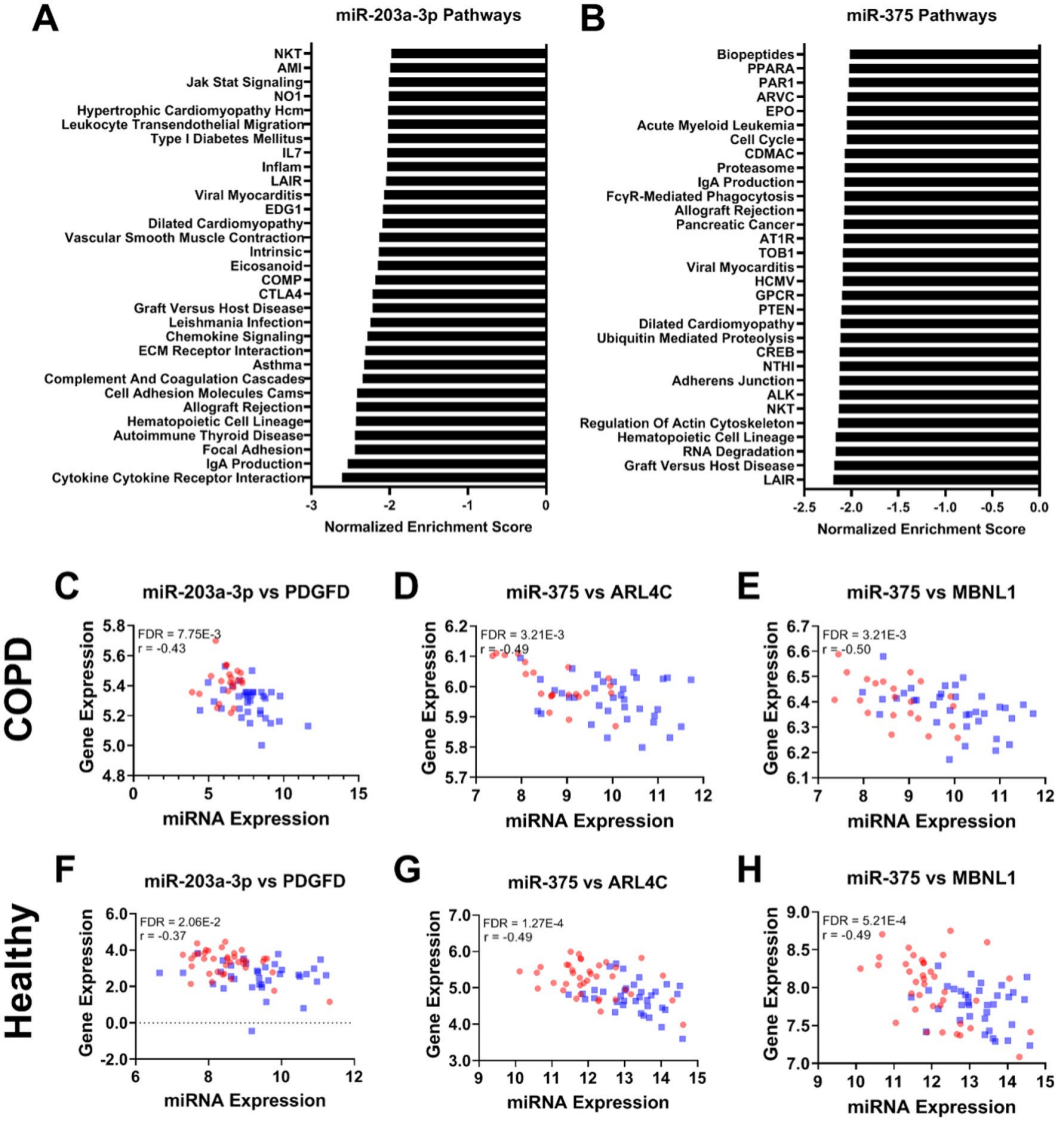
Significant pathway negatively enrichment by miRNA-203a-3p and miR-375 and correlation of aforementioned miRNAs with genes in both COPD patients as well as respiratory healthy controls, excluding correlations that were considered not biologically relevant due to a low r-value. (**A**) Enriched pathways affected by miR-203a-3p in COPD patients. (**B**) The enriched pathways affected by miR-375 in COPD patients. Scatter plots (**C**–**E**) show the correlation between miRNA expression and gene expression levels in COPD patients for all significant negatively correlated predicted targets; miR-203a-3p and *PDGFD*, miR-3758, and *ARL4C*, and miR-375 expression and *MBNL1*, respectively. Smoking participants are shown in blue, ex-, and non-smokers are shown in red. Scatter plots (**F**–**H**) show the correlation between miRNA expression and gene expression levels in respiratory healthy controls between miR-203a-3p and *PDGFD*, miR-375, and *ARL4C*, and mir-375 and *MBNL1*, respectively.

For the first analysis, we examined whether miR-375, miR-203a-3p and miR-31-3p affected the expression of mRNA transcripts in each study separately and then performed a meta-analysis for the miRNAs present in both studies (miR-375 and miR-203a-3p). Here, we identified 983 significant negatively correlated genes for miR-203a-3p (FDR < 0.05), and 2254 positively correlated genes. We found 2442 significantly negatively correlated genes for miR-375 and 865 positively correlated genes, and we found 1106 significant negative correlations for miR-31-3p and 1029 positive correlations (Table S1). The top 3 most strongly correlated genes (*PDGFD*, *ARL4C* and *MBNL1*) of both miR-375 and miR-203a-3p are shown in Fig. 3C–H. Gene functions are described in Table 3.

**Table 3:** Selective literature of predicted target genes that were negatively correlated to miR-203a-3p, miR-375 and miR-31-3p, whilst significant after our meta-analysis (miR-203a-3p and miR-375) or FDR significant (miR-31-3p) after correlation.

miRNA id	Gene symbol	r (COPD)	r (Healthy)	Meta-analysis FDR	Gene function
miR-375	*ARL4C*	− 0.49	− 0.52	1.45E−06	Involved in tumorigenesis in lung^41,42^
miR-375	*MBNL1*	− 0.50	− 0.49	5.52E−06	Tumorigenesis via an isoform^43^, regulates alternative splicing^44^
miR-375	*SLC16A2*	− 0.30	− 0.53	3.71E−05	Transportation of diidothyronine, thyroxine and triiodothyronine^45^
miR-375	*RLF*	− 0.35	− 0.45	2.75E−04	Tumorgenesis^46^
miR-203a-3p	*PDGFD*	− 0.43	− 0.37	9.58E−04	Fibroblast proliferation and survival, associated with adrenal suppression^32,33^
miR-375	*TCF12*	− 0.34	− 0.39	1.86E−03	Tumorgenesis in several cancers. Cell differentiation, repression of E-cadherin, cell development, differentiation of lymphocytes^47–51^
miR-375	*WBP1L*	− 0.25	− 0.43	2.20E−03	Regulates CXCR4 signalling in leukocytes and alters B-cell development^36^
miR-375	*PDE5A*	− 0.49	− 0.29	3.13E−03	Regulates cyclic GMP, intercellular messengers that mediate the effects of extracellular signalling molecules^52,53^. Regulates pulmonary hypertension
miR-375	*APBB2*	− 0.42	− 0.32	3.72E−03	Adapter protein, signal transduction^54,55^
miR-375	*ZNF385D*	− 0.54	− 0.24	4.87E−03	
miR-375	*CNIH4*	− 0.39	− 0.33	5.13E−03	Located in the secretory pathway, it promotes the exit of GPCRs^56^
miR-375	*MMD*	− 0.45	− 0.27	7.77E−03	Macrophages activation^57^, highly expressed in non-small cell lung cancer^58^
miR-375	*HAS2*	− 0.40	− 0.29	9.63E−03	Regulates cell adhesion, extracellular matrix formation, tumorigenesis^59,60^
miR-375	*CXCL12*	− 0.39	− 0.29	1.03E−02	Chemoattractant for T-lymphocytes and monocytes^61^
miR-375	*LDHB*	− 0.41	− 0.27	1.35E−02	Involved in tumorigenesis^62^, a subunit of lactate dehydrogenase
miR-375	*TRAPPC6B*	− 0.28	− 0.29	3.73E−02	Vesicle transport^63^, involved in the secretory pathway^64^
miR-375	*APBB1IP*	− 0.33	− 0.26	4.02E−02	Adapter protein, signal transduction^54,55^
miR-203a-3p	*EBF3*	− 0.58	− 0.10	4.22E−02	B-cell differentiation, bone development, neurogenesis and tumour suppressor via cell cycle arrest and apoptosis^34^
				**FDR**	
miR-31-3p	*NFATC2*	− 0.58		2.74E−06	nuclear factor of activated T cells—translocates to the nucleus upon T cell receptor (TCR)—immune response^65,66^
miR-31-3p	*PLCB1*	− 0.50		8.61E−05	intracellular transduction of many extracellular signals
miR-31-3p	*PDE5A*	− 0.48		1.40E−04	cAMP binding—smooth muscle relaxation in the cardiovascular system^67,68^
miR-31-3p	*RXFP1*	− 0.48		1.43E−04	
miR-31-3p	*DLC1*	− 0.47		2.52E−04	GAP family proteins participate in signalling pathways that regulate cell processes involved in cytoskeletal changes^69^.—angiogenesis^70^
miR-31-3p	*TOX*	− 0.45		4.06E−04	HMG box DNA binding domain.—chromatin assembly, transcription and replication^71^—T-cell development^72^
miR-31-3p	*C1QTNF7*	− 0.45		4.54E−04	
miR-31-3p	*PIK3CG*	− 0.42		1.15E−03	immune response, proliferation and survival^73,74^
miR-31-3p	*IL15*	− 0.40		1.98E−03	regulates T and natural killer cell activation and proliferation^75^
miR-31-3p	*DIRAS3*	− 0.40		2.24E−03	Inhibits RAS/MAPK signalling^76^
miR-31-3p	*WASF3*	− 0.38		4.02E−03	transduce signals that involve changes in cell shape, motility or function^77^
miR-31-3p	*LGI2*	− 0.37		4.53E−03	
miR-31-3p	*KCNIP2*	− 0.37		4.54E−03	Voltage-gated potassium channels
miR-31-3p	*TMOD2*	− 0.36		5.48E−03	actin regulatory protein^77^
miR-31-3p	*RAB3C*	− 0.35		8.29E−03	

### Correlation with predicted target genes

For the targeted analysis, we focused on negatively correlated predicted targets for miR-203a-3p, miR-375 and miR-31-3p. First, predicted targets were identified using miRNAtap, and then the overlap was taken from the negatively correlated genes from the previous analysis.

Of the 983 genes negatively correlated with miR-203a-3p, two genes were also predicted targets; *PDGFD*, previously associated with fibroblast proliferation and survival, and *EBF3,* previously associated with B cell differentiation and cell survival through apoptosis and cell cycle arrest^32–34^.

Of the 2442 genes negatively correlated to miR-375, 18 were also predicted targets; *ZNF385D*, *MBNL1*, *ARL4C*, *PDE5A*, *MMD*, *APBB2*, *LDHB*, *HAS2*, *CXCL12*, *CNIH4*, *APBB1IP*, *DTHD1*, *LST1*, *RLF*, *SLC16A2*, *TCF12*, *TRAPPC6B* and *WBP1L* (Fig. 3C–H).

For miR-31-3p, we found that 14 of the 1106 negatively correlated genes were also predicted targets; *NFATC2, PLCB1, PDE5A, RXFP1, DLC1, TOX, C1QTNF7, PIK3CG, IL15, DIRAS3, WASF3, LGI2, KCNIP2* and *TMOD2*.

### Pathways affected by miRNA expression that change with current smoking patients

To investigate the pathways influenced by changes in miRNA levels, we performed pathway analysis on (1) all genes of which the expression correlated to the identified miRNAs, for a global effect (Fig. 3A,B), and (2) the significantly negatively correlated predicted targets of the miRNAs, for a direct effect of these miRNAs.

For miR-203a-3p, we found a higher expression associated with genes involved in cellular protection (xenobiotic and glutathione metabolism pathways), cellular repair (cell cycle, citrate cycle pathways), and protease pathway, as well as negative associations with pro-inflammatory pathways. In addition, the MAPK signalling pathway was negatively associated with miR-203a-3p associated genes in COPD patients (Table 4).

**Table 4:** Significant gene sets negatively affected by the identified miRNAs in COPD patients and asymptomatic participants. Only the top pathways with a family-wise error rate < 0.05 in the COPD patients are shown.

miRNA	Gene sets	COPD			Asymptomatic			Total genes
		NES	FWER P-value	Core-enriched genes	NES	FWER P-value	Core-enriched genes	
miR-203a-3p	Cytokine–cytokine Receptor interaction	− 2.58	0.00E+00	122	− 2.78	0.00E+00	125	253
	Intestinal immune Network For IgA production	− 2.53	0.00E+00	36	− 2.92	0.00E+00	26	45
	Focal adhesion	− 2.46	0.00E+00	78	− 2.27	8.00E−03	88	197
	Systemic lupus Erythematosus	− 2.43	0.00E+00	27	− 2.96	0.00E+00	31	50
	Autoimmune thyroid disease	− 2.43	0.00E+00	31	− 2.71	0.00E+00	22	49
	Hematopoietic cell lineage	− 2.42	0.00E+00	51	− 2.80	0.00E+00	49	83
	Asthma	− 2.42	0.00E+00	14	− 2.54	0.00E+00	14	28
	Cell adhesion Molecules cams	− 2.41	0.00E+00	60	− 2.58	0.00E+00	61	126
	Allograft rejection	− 2.41	0.00E+00	26	− 2.99	0.00E+00	23	35
	Complement and coagulation cascades	− 2.32	0.00E+00	26	− 2.18	1.60E−02	36	67
	ECM receptor interaction	− 2.29	0.00E+00	40	− 2.04	4.20E−02	43	83
	Chemokine signaling	− 2.27	0.00E+00	72	− 2.21	1.30E−02	53	179
	Leishmania infection	− 2.25	0.00E+00	37	− 2.84	0.00E+00	26	65
miR-375	Ribosome	− 3.05	0.00E+00	53	− 2.72	0.00E+00	65	75
	Leishmania infection	− 2.75	0.00E+00	35	− 1.93	1.20E−02	38	65
	Focal adhesion	− 2.74	0.00E+00	105	− 2.04	0.00E+00	103	197
	Il2rb	− 2.71	0.00E+00	25	− 2.20	0.00E+00	26	37
	MET	− 2.66	0.00E+00	25	− 2.08	0.00E+00	22	33
	B cell receptor signaling	− 2.62	0.00E+00	44	− 1.84	1.03E−01	40	75
	PDGF	− 2.60	0.00E+00	20	− 1.94	1.20E−02	23	28
	GH	− 2.58	0.00E+00	16	− 2.13	0.00E+00	20	27
	FCER1	− 2.58	0.00E+00	25	− 1.93	1.20E−02	29	39
	GLEEVEC	− 2.51	0.00E+00	18	− 1.94	1.10E−02	15	23
	Renal cell carcinoma	− 2.51	0.00E+00	37	− 2.03	1.00E−03	39	66
	TCR	− 2.49	0.00E+00	29	− 1.98	3.00E−03	33	44
	CTCF	− 2.48	0.00E+00	18	− 2.05	0.00E+00	15	24
	Integrin	− 2.46	0.00E+00	22	− 2.06	0.00E+00	21	34
miR-31-3p	Hematopoietic cell lineage	− 2.56	0.00E+00	44				83
	Intestinal immune network For IgA production	− 2.56	0.00E+00	24				46
	Cytokine–cytokine Receptor interaction	− 2.55	0.00E+00	110				254
	Systemic lupus Erythematosus	− 2.46	0.00E+00	29				50
	Focal adhesion	− 2.44	0.00E+00	74				197
	Leishmania infection	− 2.43	0.00E+00	28				66
	ECM receptor interaction	− 2.39	0.00E+00	45				83
	Cell adhesion Molecules cams	− 2.31	0.00E+00	51				128
	Chemokine signaling Pathway	− 2.28	0.00E+00	65				180
	Autoimmune thyroid disease	− 2.26	0.00E+00	35				49
	Asthma	− 2.26	0.00E+00	18				28
	Allograft rejection	− 2.25	0.00E+00	28				35
	LAIR pathway	− 2.24	0.00E+00	13				16

*NES* normalized enrichment score, *FWER P-value* family-wise error rate p-value.

We found a global positive effect of genes associated with a higher expression of miR-375 on olfactory transduction, glycan biosynthesis and linoleic acid metabolism, and adverse effects on pro-inflammatory pathways in COPD patients, for example, via the cytokine-cytokine receptor integration pathway (Table 4).

A higher expression of miR-31-3p had positive associations with genes involved in pathways related to xenobiotic metabolism, cellular repair and linoleic acid metabolism, as well as negative associations with genes involved in pro-inflammatory pathways.

### Relation between cell-type composition and genes associated with miRNAs

Two methods were used to assess potential effects of cell-type composition on the expression of the miRNA-associated genes, i.e. GSVA based on single-cell gene signatures derived from a publically available dataset and correlation analysis with inflammatory cell counts assessed in adjacent biopsies of the same patients.

With the GSVA analysis, we found a significant negative correlation between miR-203a-3p expression and cell type composite scores of single-cell signatures for fibroblasts, luminal macrophages, endothelial cells, mast cells, smooth muscle cells, B-cells, submucosal cells and inflammatory dendric cells. In contrast, we found a positive association of this miRNA with proliferating basal cells and club and goblet cells with current smoking in both COPD patients and respiratory healthy controls.

For miR-375, we found a negative correlation with cell type composite scores for endothelial cells, smooth muscle cells, mast cells, fibroblasts, basal cells and luminal macrophages., and a positive correlation with club and goblet cells, ionocytes and submucosal cells in both studies (Supplementary Tables S2 and S3). Activated endothelium, fibroblasts, luminal macrophages, mast cells and smooth muscle cells GSVA composite scores were negatively associated with mir-31-3p while proliferating basal cells and club and goblet cells were positively associated.

In addition, we correlated the miRNA expression profiles to cell counts of eosinophils, neutrophils, macrophages, mast cells, CD3 + , CD4 + or CD8 + T-cells, CD20 + B-cells, and percentage of PAS staining positive mucus in the COPD study. We found a negative correlation between eosinophils and miR-203a-3p expression and a positive correlation between miR-31-3p and epithelial cells, but no association with miR-375 (data not shown).

## Discussion

In the current study, we identified two miRNAs (miR-203a-3p and miR-375) that are significantly higher expressed with current smoking in COPD patients and respiratory healthy subjects, indicating a common effect of smoking regardless of disease status. Additionally, we found that miR-31-3p is associated with current smoking only in COPD patients.

We found that miR-375 is higher expressed in bronchial biopsies of current-smoking COPD patients and respiratory healthy controls, and we validated this finding in an independent dataset. We identified 18 predicted target genes that decrease with increased miR-375 expression. These included *TCF12, WBP1L, CXCL12, MMD, and LST1* that were previously associated with inflammation via T-cell precursor differentiation^35^, altering B-cell development^36^, chemotaxis^57^, differentiation of monocytes to macrophages^53^, or differentiation of other cells^59^. These predicted target genes were not enriched in one specific pathway. However, our global miRNA pathway analysis showed that genes associated with miR-375 are lower expressed in pro-inflammatory pathway activity, such as the IL2RB and B-cell receptor signalling pathway. We continued our investigation using cellular deconvolution and found that higher levels of miR-375 are associated with lower levels of basal cells, fibroblasts, mast cells, smooth muscle cells, endothelial cells and luminal macrophages and higher levels of secretory cells, submucosal cells and ionocytes. In contrast, no association was found with inflammatory cell populations as determined histologically. As we consistently find miR-375 being increased by smoke across all datasets and a positive correlation between miR-375 with secretory cells, this may indicate cell-specific expression in secretory cells as it is well established that the levels of secretory cells are higher in current smokers^37^. The high number of genes and pathways related to inflammation changed by miR-375, but the lack of correlation to gene signatures typical for inflammatory cells might indicate an increased activation of inflammatory cells caused by miR-375 rather than a change in cell numbers or composition.

We showed that miR-203a-3p is more highly expressed in bronchial biopsies of current- compared to ex-smokers with COPD and in current- versus non-smoking respiratory healthy subjects. Furthermore, two negatively correlated predicted targets of miR-203a-3, *EBF3* and *PDGFD,* were involved in cell cycle arrest and apoptosis^31^ and fibroblast proliferation^29,30^, respectively. Therefore, the lower *EBF3* and *PDGFD* in response to smoking in association with miRNA-203a-3p by smoking potentially indicates a protective response and stimulation of repair as a response to cigarette smoke. In addition, we found that genes associated with xenobiotic metabolism were positively correlated with miR-203a-3p, suggesting that this miRNA facilitates the metabolism of harmful particles in cigarette smoke^38^. Additionally, we found that genes involved in pro-inflammatory pathways (e.g. cytokine-cytokine receptor interaction, IgA production and chemokine signalling) were negatively correlated with miR-203a-3p, suggesting an anti-inflammatory effect for this miRNA. We associated these microRNAs with cell signatures to assess whether a change in cell populations may drive the smoking-induced increase in miR-203a-3p expression. We found higher smoking-induced expression of miR-203a-3p associated with lower proportions of fibroblasts, luminal macrophages, endothelial cells, mast cells, smooth muscle cells, B-cells, inflammatory dendritic cells, and submucosal cells and higher proportions of secretory cells (club and goblet cells) and proliferating basal cells. Therefore, it could be speculated that miR-203a-3p might play a role in the basal cell's differentiation towards secretory cells, leading to more mucus production in smokers or that this miRNA is selectively expressed in secretory cells. This, however, has now only been demonstrated in biopsies and requires further investigation in isolated cells to see the relationship of this miRNA with basal cell differentiation towards secretory cells, with and without smoke exposure.

MiR-31-3p was higher in current- compared to ex-smoking COPD patients, and this miRNA was not found in the respiratory healthy control dataset. We found 14 predicted target genes that decreased with an increase of miR-31-3p. Among these genes was *PDE5A*, which was involved in smooth muscle contraction and relaxation, as well as cell proliferation, cell signalling and included pro-inflammatory w Inhibition of *PDE5A* is involved in preventing tobacco smoke-induced emphysema^39^, suggesting a protective role of this miRNA in current smokers with COPD as an attempt to limit the harmful effects of smoking^40^. In a previous study, it was shown that *PDE5A* was decreased after smoke exposure in lung tissue of healthy individuals^40^. The higher expression of miR-31-3p could suppress *PDE5A* expression even further in patients with COPD.

There were some limitations to the current study. As the miRNAs expression in the healthy group were conducted on different platforms, these miRNAs were not directly comparable. This may have led to the lack of expression of miRNA-31-3p in the healthy cohort. However, as the healthy cohort was conducted with small-RNA-Seq, it should have been picked up when present, and therefore we are confident in the current results. As a strength of the study, we were able to directly correlate the miRNA to matched expression data in the same subject allowing for the identification of direct miRNA-target gene interactions. Predicted targets for the miRNAs were identified using miRNAtap, which relies on algorithms and databases. However, as these are only predictions and not necessarily accurate, having the paired gene expression and miRNA expression needs additional confirmation of the associations.

In conclusion, we found two miRNAs, miR-203a-3p and miR-375, that are upregulated in bronchial biopsies of smokers compared to ex- and non-smokers in both COPD and respiratory healthy controls. These miRNAs play a role in the detoxification and inflammatory response to smoke response. In addition, we identified that miR-31-3p was upregulated only in current smokers versus ex-smokers with COPD. miR-31-3p might have a protective role via decreasing *PDE5A* expression and thus smoke-induced emphysema. We propose that our identified miRNAs are candidates for future studies aimed at unravelling which smoking-induced inflammatory mechanisms are mainly reactive and which are involved in COPD pathogenesis.

## Supplementary Information

Supplementary Information 1.

Supplementary Information 2.
