# Supplementary material for: High miR203a-3p and miR-375 expression in the airways of smokers with and without COPD
Source: Sci Rep. 2022 Apr 4;12:5610. doi: 10.1038/s41598-022-09093-0 (PMC8980043; doi:10.1038/s41598-022-09093-0)
Supplement: Supplementary file 2 — Supplementary Information 2. [file 41598_2022_9093_MOESM2_ESM.docx]

**Table S1. Correlations of miR-203q-3p and miR-375 to genes in both the COPD and respiratory healthy dataset.** Meta-analysis is performed between the datasets.

(See Table S1.csv)

**Table S2. Significance of enrichment scores for single-cell signatures in named cell types.**

|  | **COPD** | | | **Asymptomatic** | | |
| --- | --- | --- | --- | --- | --- | --- |
| **Cell Type** | **P-value** | **T-Statistic** | **Standard Error** | **P-value** | **T-Statistic** | **Standard Error** |
| Activated endothelium | 6.64E-03 | 2.86 | 0.13 | 7.98E-01 | 0.26 | 0.11 |
| All Basal cells | 8.43E-02 | 1.76 | 0.08 | 2.79E-02 | 2.25 | 0.08 |
| B cells | 7.55E-03 | 2.79 | 0.13 | 5.47E-01 | 0.61 | 0.12 |
| Proliferating Basal cells | 4.60E-05 | -4.49 | 0.12 | 2.30E-05 | -4.54 | 0.12 |
| Ciliated cells | 1.64E-01 | 1.42 | 0.12 | 4.54E-01 | 0.75 | 0.10 |
| Club and Goblet cells | 5.96E-03 | -2.93 | 0.09 | 2.53E-10 | -7.41 | 0.08 |
| Fibroblasts | 1.38E-03 | 3.41 | 0.12 | 1.72E-01 | 1.38 | 0.13 |
| Inflammatory DCs | 8.82E-02 | 1.74 | 0.11 | 3.64E-01 | -0.91 | 0.13 |
| Ionocytes | 1.64E-02 | -2.52 | 0.13 | 2.44E-07 | -5.79 | 0.10 |
| Luminal macrophages | 2.59E-02 | 2.30 | 0.15 | 6.99E-02 | 1.84 | 0.15 |
| Mast cells | 3.91E-03 | 3.03 | 0.14 | 6.75E-01 | 0.42 | 0.13 |
| Neutrophils | 3.65E-01 | 0.92 | 0.15 | 2.58E-01 | 1.14 | 0.15 |
| Smooth muscle | 8.84E-03 | 2.74 | 0.13 | 1.39E-02 | 2.52 | 0.13 |
| Submucosal | 5.05E-01 | -0.67 | 0.14 | 8.27E-02 | -1.76 | 0.13 |

**Table S3. Enrichment scores of single-cell signatures within named cell types correlated with expression values of miRNAs.**

|  | **COPD** | | | **Asymptomatic** | | |
| --- | --- | --- | --- | --- | --- | --- |
| **Cell Type** | **r** | **T-Statistic** | **FDR** | **r** | **T-Statistic** | **FDR** |
| **miR-203a-3p** |  |  |  |  |  |  |
| Activated endothelium | -0.53 | -4.69 | 2.63E-04 | -0.24 | -2.07 | 7.38E-02 |
| All Basal cells | -0.22 | -1.69 | 1.50E-01 | 0.05 | 0.41 | 7.34E-01 |
| B cells | -0.24 | -1.80 | 1.37E-01 | -0.34 | -3.08 | 7.63E-03 |
| Proliferating Basal cells | 0.13 | 0.97 | 3.85E-01 | 0.48 | 4.57 | 2.82E-04 |
| Ciliated cells | 0.12 | 0.93 | 3.85E-01 | -0.11 | -0.97 | 4.25E-01 |
| Club and Goblet cells | 0.30 | 2.33 | 5.44E-02 | 0.43 | 4.02 | 9.98E-04 |
| Fibroblasts | -0.49 | -4.22 | 6.43E-04 | -0.20 | -1.75 | 1.32E-01 |
| Inflammatory DCs | -0.18 | -1.38 | 2.19E-01 | -0.08 | -0.67 | 5.89E-01 |
| Ionocytes | 0.20 | 1.49 | 1.98E-01 | 0.33 | 2.92 | 9.43E-03 |
| Luminal macrophages | -0.37 | -2.99 | 1.46E-02 | -0.34 | -3.04 | 7.63E-03 |
| Mast cells | -0.33 | -2.60 | 3.36E-02 | -0.15 | -1.25 | 3.02E-01 |
| Neutrophils | -0.02 | -0.16 | 8.73E-01 | -0.38 | -3.46 | 3.18E-03 |
| Smooth muscle | -0.47 | -3.94 | 1.08E-03 | -0.38 | -3.50 | 3.18E-03 |
| Submucosal | -0.24 | -1.83 | 1.37E-01 | -0.02 | -0.20 | 8.40E-01 |
| **miR-375** |  |  |  |  |  |  |
| Activated endothelium | -0.57 | -5.18 | 4.62E-05 | -0.14 | -1.19 | 3.35E-01 |
| All Basal cells | -0.30 | -2.35 | 3.89E-02 | -0.53 | -5.28 | 1.85E-05 |
| B cells | -0.21 | -1.63 | 1.41E-01 | -0.03 | -0.29 | 7.71E-01 |
| Proliferating Basal cells | 0.11 | 0.84 | 4.69E-01 | 0.08 | 0.68 | 5.81E-01 |
| Ciliated | 0.04 | 0.28 | 8.38E-01 | 0.14 | 1.21 | 3.35E-01 |
| Club and Goblet cells | 0.49 | 4.13 | 4.30E-04 | 0.37 | 3.38 | 5.10E-03 |
| Fibroblasts | -0.41 | -3.32 | 3.75E-03 | -0.35 | -3.15 | 5.96E-03 |
| Inflammatory DCs | -0.21 | -1.62 | 1.41E-01 | -0.08 | -0.70 | 5.81E-01 |
| Ionocytes | 0.48 | 4.02 | 4.99E-04 | 0.37 | 3.31 | 5.10E-03 |
| Luminal macrophages | -0.34 | -2.64 | 2.16E-02 | -0.06 | -0.49 | 6.75E-01 |
| Mast cells | -0.50 | -4.27 | 3.62E-04 | -0.35 | -3.13 | 5.96E-03 |
| Neutrophils | 0.02 | 0.17 | 8.63E-01 | -0.20 | -1.69 | 1.67E-01 |
| Smooth muscle | -0.51 | -4.36 | 3.62E-04 | -0.32 | -2.84 | 1.18E-02 |
| Submucosal | 0.30 | 2.30 | 3.93E-02 | 0.46 | 4.40 | 2.58E-04 |
| **miR-31-3p** |  |  |  |  |  |  |
| Activated endothelium | -0.46 | -3.81 | 1.25E-03 |  |  |  |
| All Basal cells | -0.06 | -0.48 | 6.36E-01 |  |  |  |
| B cells | -0.27 | -2.09 | 7.17E-02 |  |  |  |
| Proliferating Basal cells | 0.37 | 2.99 | 1.17E-02 |  |  |  |
| Ciliated | -0.08 | -0.57 | 6.16E-01 |  |  |  |
| Club and Goblet cells | 0.33 | 2.58 | 2.53E-02 |  |  |  |
| Fibroblasts | -0.46 | -3.85 | 1.25E-03 |  |  |  |
| Inflammatory DCs | -0.19 | -1.44 | 1.96E-01 |  |  |  |
| Ionocytes | 0.12 | 0.93 | 4.16E-01 |  |  |  |
| Luminal macrophages | -0.48 | -4.07 | 1.25E-03 |  |  |  |
| Mast cells | -0.35 | -2.73 | 1.98E-02 |  |  |  |
| Neutrophils | -0.25 | -1.95 | 8.85E-02 |  |  |  |
| Smooth muscle | -0.46 | -3.89 | 1.25E-03 |  |  |  |
| Submucosal | -0.23 | -1.72 | 1.27E-01 |  |  |  |
| FDR: False discovery rate | | | | | | |

**Table S4. Differential expression analysis of miRNAs in smokers compared to ex-smokers in an independent dataset.**

|  | Smoking Status | | COPD | |
| --- | --- | --- | --- | --- |
|  | T statistic | ANOVA | T statistic | ANOVA |
| miR-203 | -0.55 | 5.87E-01 | 0.31 | 7.60E-01 |
| miR-375 | 6.22 | 7.58E-08 | 1.70 | 9.55E-02 |
| miR-31-3p | 1.05 | 3.00E-01 | 1.34 | 1.86E-01 |

|  | Smoking Status  COPD | | Smoking Status  Non-COPD | |
| --- | --- | --- | --- | --- |
|  | T statistic | ANOVA | T statistic | ANOVA |
| miR-203 | -0.03 | 9.74E-01 | 1.05 | 3.03E-01 |
| miR-375 | -2.86 | 8.49E-03 | -5.86 | 4.15E-06 |
| miR-31-3p | -0.65 | 5.22E-01 | 0.97 | 3.39E-01 |
